# Microarray analysis after RNA amplification can detect pronounced differences in gene expression using limma

**DOI:** 10.1186/1471-2164-7-252

**Published:** 2006-10-09

**Authors:** Ilhem Diboun, Lorenz Wernisch, Christine Anne Orengo, Martin Koltzenburg

**Affiliations:** 1 Bioinformatics Unit, Department of Biochemistry and Molecular Biology, University College London, Gower Street, London, WC1E 6BT, UK; 2 School of Crystallography, Birkbeck College, University of London, Malet Street, London WC1 7HX, UK; 3 Neural Plasticity Unit, UCL Institute of Child Health, 30 Guilford St, London WC1N 1EH, UK

## Abstract

**Background:**

RNA amplification is necessary for profiling gene expression from small tissue samples. Previous studies have shown that the T7 based amplification techniques are reproducible but may distort the true abundance of targets. However, the consequences of such distortions on the ability to detect biological variation in expression have not been explored sufficiently to define the true extent of usability and limitations of such amplification techniques.

**Results:**

We show that expression ratios are occasionally distorted by amplification using the Affymetrix small sample protocol version 2 due to a disproportional shift in intensity across biological samples. This occurs when a shift in one sample cannot be reflected in the other sample because the intensity would lie outside the dynamic range of the scanner. Interestingly, such distortions most commonly result in smaller ratios with the consequence of reducing the statistical significance of the ratios. This becomes more critical for less pronounced ratios where the evidence for differential expression is not strong. Indeed, statistical analysis by limma suggests that up to 87% of the genes with the largest and therefore most significant ratios (p < 10e^-20^) in the unamplified group have a p-value below 10e^-20 ^in the amplified group. On the other hand, only 69% of the more moderate ratios (10e^-20 ^< p < 10e^-10^) in the unamplified group have a p-value below 10e^-10 ^in the amplified group. Our analysis also suggests that, overall, limma shows better overlap of genes found to be significant in the amplified and unamplified groups than the Z-scores statistics.

**Conclusion:**

We conclude that microarray analysis of amplified samples performs best at detecting differences in gene expression, when these are large and when limma statistics are used.

## Background

Microarray technology offers a high throughput approach to transcriptional profiling on a genome wide scale. However, the relatively large amount of starting material required for standard hybridization has limited its full potential. In complex biological systems such as the nervous system, the utility of this approach is complicated by the fact that even in anatomically discrete regions, many divergent cell types are intermingled. It is often desirable to investigate gene expression profiles of distinct cell types and although laser microdissection provides a solution to the problem of tissue procurement, the small amount of RNA that can be harvested has precluded a straightforward combination of both technologies. This limitation is compounded by the need of replication essential for statistical analysis.

Another scenario in which the lack of sufficient tissue availability has been challenging is the correlation of the phenotype in individual experimental animals with comprehensive gene expression profiles. For example, so far it has not been possible to correlate the inter-individual behavioural variability in animal models of chronic pain with the corresponding correlates in gene expression in the principal anatomical components of the pain pathway as such structures in individual animals do not yield sufficient amounts of RNA for standard hybridization protocols.

To overcome these issues, increasingly sophisticated approaches to RNA amplification from small tissue samples have been developed for use with microarrays. These fall principally into two categories. One is based on PCR and is characterized by an exponential increase in copy number while the other is based on the T7-polymerase in-vitro transcription (IVT) to achieve a linear amplification of targets. For maximum fidelity, linearity of target amplification is desirable. Thus, substantial work has focused on exploring the T7 linear techniques [1-4], in particular the Affymetrix small sample protocol II has been assessed by numerous studies with interesting results. Analysis based on correlating intensity levels and assessing concordance in detection calls has indicated a high level of reproducibility [2,3,5,6]. However, occasional failure to maintain the true abundance level of transcripts was also found [3,5,6] due to the protocol 3'bias [1,2,4,6,7]. Such bias is thought to be related to the use of random hexamers to prime the RT reaction in the additional round of amplification. With priming that is remote from the 3'end, RT may not be successfully completed causing a diminution in the signal from the 5' regions.

These observations are useful indicators of protocol validity; though, the ultimate fidelity criterion is the ability to maintain differential expression between different tissues or under varying experimental conditions. Some previous studies have reported a 50% drop in significant changes in gene expression using RNA amplification [4], suggesting that RNA amplification may suffer major problems and is potentially unsuitable for microarray analysis.

In this study, we critically appraise the suitability and merits of transcript amplification from small biological samples for microarray expression profiling using the Affymetrix small sample protocol II. We confirm previous findings on the reproducibility and fidelity of the protocol. We present compelling evidence for the 3' bias introduced by RNA amplification. We show how distortions in intensity may be reflected in the expression ratios from biologically different samples. Importantly, we explore the effect of distortions in expression ratios on their statistical significance.

## Results

**Figure 1:**
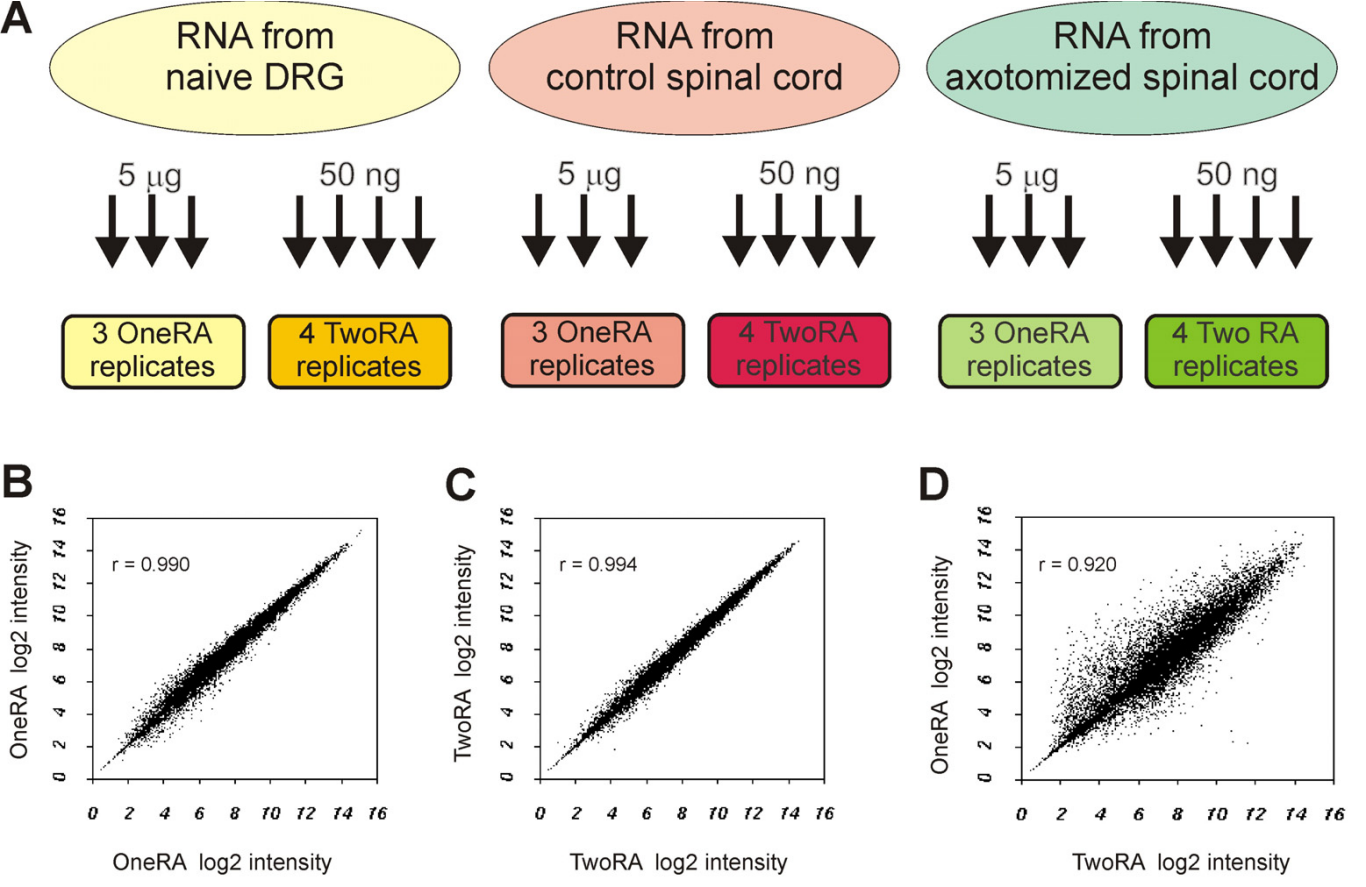
**A, experimental design. B&C&D, correlation of log2 intensities within and between protocol groups**. B&C, scatter plots of log_2 _signal intensities from a pair of replicates treated with the OneRA and the TwoRA protocol respectively. D, scatter plot of log_2 _signal intensities averaged across the OneRA replicates versus that from the TwoRA replicates. The coefficient of correlation (r) value is given for each pair. Similar results were obtained with the SN and SA sample groups.

In this study, we undertake a detailed analysis of target amplification for microarrays using the Affymetrix small sample protocol II. This analysis was performed using control data from standard protocol preparations as reference. For the rest of the article, we refer to the standard protocol as the *OneRA *(one round amplification) protocol and the small sample protocol as the *TwoRA *(two rounds amplification) protocol. TwoRA and OneRA samples from three different biological groups were used in this study: the DRG (dorsal root ganglia) group, the SN (spinal cord from normal animals) group and the SA (spinal cord from animals with axotomised sciatic nerve) group. In each group, there are 4 replicates from the TwoRA and 3 replicates from the OneRA (Figure 1a). While, the main objective of this study is to assess the extent to which biologically relevant variation in gene expression can be detected in the TwoRA, we begin by assessing the reproducibility of the TwoRA protocol and its fidelity in maintaining the relative abundance of targets.

### Reproducibility and fidelity in maintaining expression levels

Scatter plots of log2 intensities from paired TwoRA replicates show expectedly high level of consistency similar to that observed with the OneRA replicates (Figure 1b &1c) ((r) ranging from 0.990 to 0.994 among all possible pairs of the TwoRA replicates and the OneRA replicates from all biological groups). However, comparing the average log2 intensity values from the OneRA and the TwoRA (Figure 1d) for a single tissue, we see evidence of variability implying that the TwoRA protocol may distort the signal.

We used an ANOVA approach to confirm that the variability between protocol groups is greater than that among replicates within each group. In particular, a one-way, two-levels ANOVA analysis was performed for each gene separately, with 3 measurements from the OneRA (level 1) and 4 measurements from the TwoRA (level 2). First, the between group mean sum of squares *MSA *as well as the mean residual sum of squares *MSE *were calculated. The median of the MSA was higher than the median of the MSE (given in parenthesis) in all biological groups: DRG 0.050 (0.023), SN 0.062 (0.016), SA 0.068.

To test whether protocol variability is significantly greater than the residual variability, we derived p-values from the F-values (MSA/MSE) for each gene (using the upper tail of an F-distribution with 1 and 3 + 4 – 2 degrees of freedom). In fact, the p-values were far from uniformly distributed. Storey suggests the following estimate of the proportion of hypotheses from the null using p-values: the fraction of p-values above the median p-value m, divided by (1-m) [38]. This results in the following estimate of the proportion of genes with significantly higher amplification variability: DRG 47%, SN 50%, SA 41%. That is, in all cases at least 40% of genes show differences between protocols, which are not explained by variability within replicates.

### Signal distortion following TwoRA

**Figure 2:**
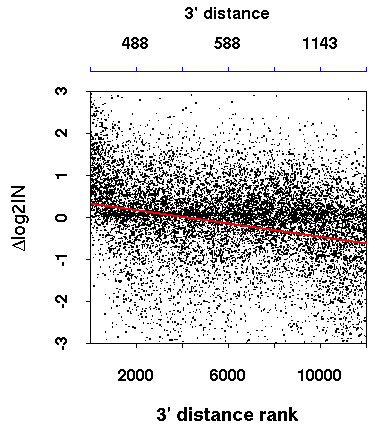
**Deviation in log2 intensity following TwoRA (Δlog2IN) as a function of probeset 3' distance rank**. Δlog2IN values on the y-axis were calculated by subtracting the mean OneRA from the mean TwoRA probeset log2 intensities. The x-axis shows the ranks of probesets locations. Probesets locations are relative to the 3' end of the transcripts (refer to methods for more details). Since the probesets locations have a skewed distribution, their ranks were plotted instead of their absolute values; this allows dispersion of data points. The actual probesets locations that correspond to the rank intervals on the x-axis are shown on the blue horizontal axis on the top of the figure. The regression line is shown in red. Only data from the DRG preparation were used, similar results were obtained with the SN and

To investigate signal distortion by the TwoRA protocol, we looked at the well-documented 3' bias feature of the protocol in the DRG dataset (similar results were obtained with the SA and SN). Bias arises from greater 3' representation of targets during TwoRA while 5' regions are often lost. This causes amplified RNA to be shorter than normal. Previously published work explored the increase in the 3'/5' signal ratios only from control genes (GAPDH, Actin) to prove that such effect occurred)[2,4,7]. In this study, we undertook a more comprehensive analysis by using information from all probesets on the array. More specifically, we correlated the deviation in log2 intensity following TwoRA (Δ***log2****IN*) – obtained by subtracting the log2 intensity in the OneRA from that of the TwoRA – with the probesets 3' locations on corresponding targets (see methods for a description of how these locations were obtained) (Figure 2). The trend suggests that probesets distal from the 3' end are more likely to endure an attenuation of signal intensity following TwoRA whilst those close to the 3' end are likely to show intensification of signal.

**Figure 3:**
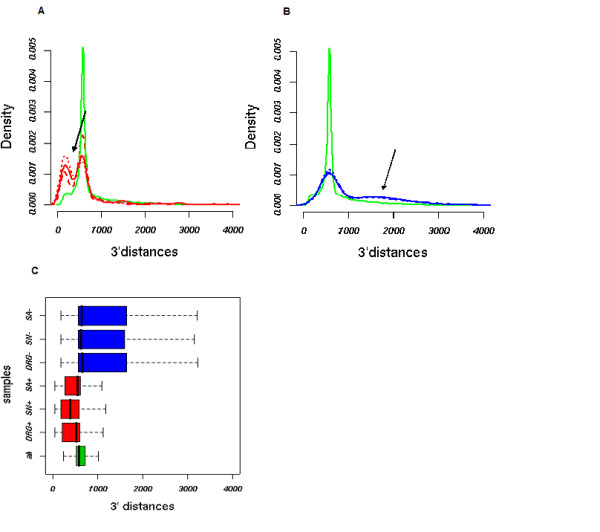
**Dist ribution of 3' distances from probesets with the most discrepant signal intensity following TwoRA (absolute Δlog2IN >= 2)**. A&B, histograms of 3' locations from the probesets at large represented on the MOE430A chip (green) versus those showing at least 2 fold change in log2 signal intensity following TwoRA (increase/decrease in signal, red/blue respectively). (Solid line), DRG. (Dashed lines), SA. (Dotted lines), SN. Arrows indicate additional peaks in the distributions apart from that of the overall population. C, a box and whisker plot showing the 0% and 100% quantiles as whiskers, the 25% and 75% quantiles as boxes and the 50% quantile as horizontal dash within the box. The plot summarises the distributions shown in A and B. One the y-axis, (+) indicates increase in signal intensity following TwoRA, (-) indicates decrease in signal intensity following TwoRA. Note that for all plots, probesets locations are in base pairs and relative to the 3' end of targets.

In a separate but related analysis, probesets whose absolute Δlog2IN values were greater than 2 were reviewed for their 3' location distribution (Figure 3). This was compared to the distribution of 3'location of all probesets on the array. The distribution for all probesets appears to be skewed and peaks at around 600 bp. The distribution of probesets with intensified signal shows an additional peak to the left suggesting a distinct population of probesets closer than average to the 3' end (Figure 3a). This is further highlighted by a decrease in the 25% quantile relative to the overall population in the boxplots on figure 3c. Conversely, the distribution from probesets with attenuated signal shows a second peak to the right indicating an overrepresentation of more distal interrogation points relative to the 3' end of targets (Figure 3b). This corresponds to an increase in the 75% quantile (Figure 3c). These results show for the first time that the most severe signal distortions following TwoRA are strongly associated with 3' positional bias.

**Figure 4:**
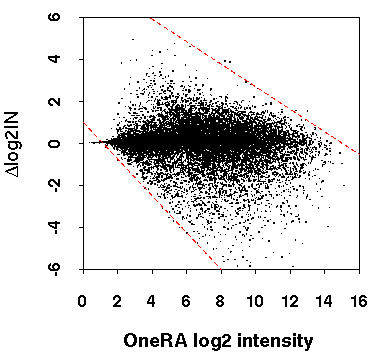
**Deviation in log2 intensity following TwoRA (Δlog2IN) versus the OneRA log2 intensity**. The dashed lines in red show roughly the limits of the intensity range, thus the upper line represents saturation while the lower line represents background noise. The analysis was done on the DRG data; similar results were obtained with the SN and SA samples.

Interestingly, with both populations of deviant probesets (Figure 3a &3b), there is a peak that overlaps with that from the distribution of the average population. This implies that among these deviant probesets, there is a population that is at the same distance from the 3' end as the general population; yet, their intensities are shifted in the TwoRA. This suggests that other factors, apart from 3'bias, are likely to be involved in distorting the signal. One possible factor is the abundance of the transcript. Thus, we correlated the deviation in log2 intensity following amplification (Δlog2IN) with the log2 intensity for the DRG, OneRA (Figure 4). The plot indicates some bias in the extreme Δlog2IN values along the intensity scale. This is due to the fact that the intensity range is limited; in other words, intensity cannot be higher than the saturation level (ceiling effect) or below background noise (floor effect).

### Fidelity in maintaining expression ratios

The ultimate aim of microarrays is the identification of differential expression. Thus, a good amplification protocol should faithfully maintain expression ratios. To verify this, we cross-compared expression ratios from biologically distinct tissue samples treated with the OneRA and the TwoRA protocols.

**Figure 5:**
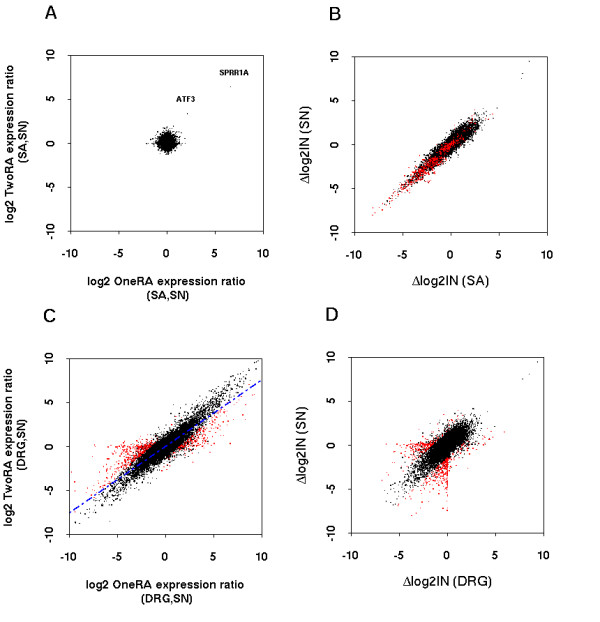
**Correlation of log2 expression ratios from the OneRA and TwoRA and that of Δlog2IN from the two compared samples**. A&C, scatter plots of log2 expression ratios from the OneRA and the TwoRA for the (SA,SN), (DRG,SN) pairs respectively. For instance, the log2 OneRA expression ratio for the (DRG,SN) pair is log2 OneRA DRG – log2 OneRA SN. For the (SN, SA) pair, the most significant differences in gene expression (ATF3 and SPRR1A) are consistent in both protocol groups. For the (DRG,SN), the ratios are more varying, the regression line (shown in blue) appears to be shifted from the diagonal towards smaller values of expression ratios in The TwoRA. B&D, scatter plots of Δlog2IN values for the (DRG,SN) pair and the (SA,SN) pair respectively. Δlog2IN were calculated by subtracting the log2 OneRA intensity from the log2 TwoRA intensity. Points in red in 'C' are probesets where the intensity in one sample could not be shifted as much as in the other sample because the intensity cannot lie outside the dynamic range of the scanner. These are referred to as FCE (floor & ceiling effect) probesets and have varying expression ratios following TwoRA. The FCE probesets also show the most varying DRG and SN Δlog2IN values (points in red, D). Though, for these same probesets, the Δlog2IN values in the SA and the SN are fairly consistent (points in red, B).

First, we considered the (SA,SN) pair. Expression ratios on log2 scale from the OneRA samples were correlated with their equivalents from the TwoRA (Figure 5a). The significant changes in expression (labelled in figure 5a) seem to be consistent in the TwoRA and the OneRA groups. These are well documented in the literature (*activating transcription factor 3 *[19] and *small proline-rich repeat protein 1A *[20]). However, there are relatively few differences in gene expression between these two biological samples, probably due to the fact that the tissue from the injured animals included areas of the spinal cord not affected by the axotomy, which could have caused a dilution of effect in the relevant areas. To reliably evaluate the effect of the TwoRA protocol on ratios, a larger profile of differential expression is needed. This was possible with the (DRG,SN) pair. Thus, we decided to base our assessment of the effect of the TwoRA on ratios on the (DRG,SN) dataset.

Encouragingly, log2 expression ratios from the (DRG,SN) treated with the OneRA and the TwoRA protocols are comparable; though they show more variability than their counterparts from the (SA,SN) pair (Figure 5c). Moreover, the regression line (shown in blue) appears to be shifted from the diagonal in a way that suggests that the expression ratios are on average slightly lower in the TwoRA relative to the OneRA.

### Variation in ratios

From our previous analysis, we know that the TwoRA protocol may shift the absolute intensity levels. However, this only affects expression ratios if the intensity is shifted unequally in the two biological samples. That is, deviations in intensity (Δlog2IN) following TwoRA, that differ in the two samples, can result in variability in the expression ratios from the OneRA and the TwoRA groups.

**Figure 6:**
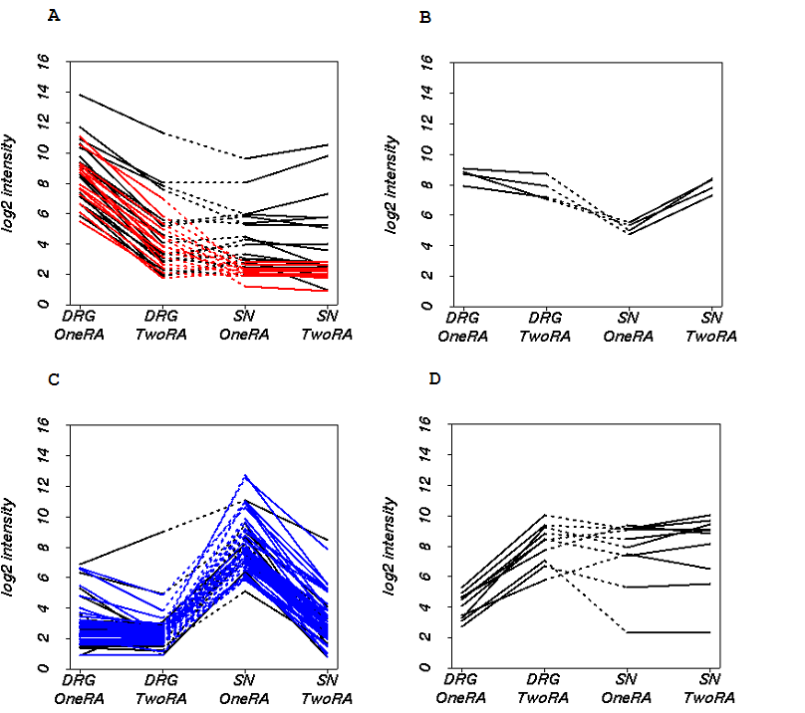
**Intensity profiles of probesets with top 100 most deviant expression ratios following TwoRA**. The profiles are classified into four categories: A&C, the intensity is reduced in the tissue sample where the gene is more expressed (DRG, SN respectively). B&D, the intensity is increased in the sample where the gene is less expressed (SN, DRG respectively). Solid lines mark the shift in intensity from OneRA to TwoRA for one tissue sample. Dashed lines link the intensity data for equivalent probesets in the two biological samples. In colour are probesets with absent call 'A' in the SN (red) and DRG (blue).

To get further insights into how unequal shifts in the intensity level following amplification of different biological samples affect the expression ratios, we ranked probesets by the absolute difference in their OneRA and TwoRA log2 expression ratios and selected the top 100 for further analysis (partly listed in Table 1). Specifically, we examined the intensities of these selected probesets across all four groups: the OneRA and the TwoRA DRG, SN. The resulting intensity profiles were classified into four categories depending on the direction of change in intensity and the tissue where this change occurred (Figure 6). The most populated categories show a significant reduction in the intensity in one of the samples whilst the intensity in the other sample is minimally reduced (Figure 6a &6c). Less frequently, the intensity increases with amplification in one of the samples but not in the other sample (Figure 6b &6d).

Interestingly, with all four categories, expression ratios are reduced following TwoRA. Moreover, the majority of the selected probesets have varying intensity levels in the DRG versus SN, OneRA. Frequently these genes are absent in one sample but highly expressed in the other sample (shown as coloured lines in figure 6), which may explain the deviation in expression ratios following TwoRA. If one takes the example of HipK2, the log2 intensity in the SN was reduced from 8.20 in the OneRA to 0.73 in the TwoRA. However, HipK2 is absent in the DRG (the OneRA log2 intensity is 0.87), thus an equivalent reduction in the intensity level in this sample is not possible (floor effect). As such the log2 expression ratio is shifted from -7.33 in the OneRA to 0.15 in the TwoRA. Alternatively, in other cases, if amplification increases the intensity in one sample, an equal shift in the other sample would not be possible if the intensity was close to saturation (ceiling effect).

Thus, distortions in the expression ratios may occur when a shift in intensity (Δlog2IN) in one sample cannot be mirrored in the other sample because it would cause the intensity to fall outside the dynamic range of the scanner. This relates to the previous analysis presented in Figure 4. Certainly, this phenomenon does not apply to genes with similar intensity levels in the two samples since a shift in intensity in one sample is equally possible in the other sample.

To assess the extent to which this phenomenon explains the deviation in expression ratios observed across the whole set of targets on the chip, we undertook an alternative analysis. We selected all probesets where a shift in intensity following TwoRA in one sample would cause the intensity in the other sample to fall outside the intensity range, that is below the background noise or higher than the saturation level. These limits were chosen to be the 3% and 98% quantiles of the distribution of signal intensity on the chip, respectively. The analysis was conducted by first determining the maximum Δlog2IN (log2 TwoRA – log2 OneRA) across the two samples for each gene. Thus, if the maximum Δlog2IN is found in sample A, we add the same Δlog2IN to the OneRA log 2 intensity from sample B. If the resulting value is outside the chosen limits, the probeset is selected by our analysis.

Since the selected probesets suffer from a **f**loor and **c**eiling **e**ffect, we shall refer to them as *FCE probesets *for the rest of the article. Clearly, the FCE probesets are the same probesets that show the most deviant expression ratios following TwoRA (coloured in red, Figure 5c) and correspondingly the most pronounced variation in shifts in intensity (Δlog2IN) in the DRG and SN samples (in red, Figure 5d). In fact, the correlation in the (DRG,SN) expression ratios across protocols (r) = 0.89 is improved to 0.93 when the FCE probesets are excluded. Interestingly, we found that the FCE probesets show consistent Δlog2IN following TwoRA in the SA and SN groups (in red, Figure 5b). This is because their intensities are similar in the (SA,SN) but different in the (DRG,SN) datasets.

### Similarity in inferring significance in ratios

The primary aim of a microarray experiment is to detect significant changes in gene expression. Our results suggest that ratios are most commonly reduced among the genes with large expression ratios in the OneRA, thus likely to be differentially expressed. Indeed, we found good evidence from the literature to suggest that 9 genes of those with the 10 most discrepant ratios following TwoRA are indeed differentially expressed between the SN and DRG (Table 2). Despite reductions in the ratios, genes can remain significant following TwoRA if their ratios are still large relative to the average population in the TwoRA. Moreover, among the population of genes with high expression ratios in the OneRA (Figure 5c), many do maintain their ratios in the TwoRA, most likely due to a faithful TwoRA of the transcripts in the two biological samples.

We applied two different statistical tests (the Z-scores and limma) to identify transcripts differentially expressed in the (DRG,SN) tissue samples prepared with both protocols (One RA and TwoRA). With each statistical method, an FDR based multiple testing correction was used and genes were ranked by their FDR values in ascending order. To measure the extent of agreement between protocols over genes identified as significant, we defined *the significance similarity score *(or the *SSS*) as the proportion of genes common to subsets of highly ranked genes from the OneRA and the TwoRA (DRG,SN) comparisons. The results are summarised in Table 3. Higher SSS were obtained using limma compared to the Z-scores method. With limma, 87 out of the top 100 most significant genes were common to the OneRA and the TwoRA comparisons as oppose to 59 using Z-scores statistics.

**Figure 7:**
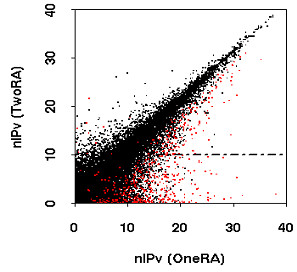
**Effect of variation in expression ratios on their statistical significance**. Scatter plots of FDR corrected nlPv (negated log transformed p-value) from the Limma analysis of the OneRA and the TwoRA DRG and SN samples. As a result of negating the p-values, large nlPv indicate stronger evidence of differential expression. Data points in red represent the FCE probesets. The dashed line is at nlPv = 10 in the TwoRA, above which genes may be considered significant.

For a more global assessment of the effect of distortions in expression ratios on their statistical significance, we used a scatter plot of negated log p-values (nlPv) from the limma analysis of the OneRA and the TwoRA (DRG,SN) (Figure 7). The FCE probesets are highlighted in red on figure 7 and it can be seen that their nlPv are least correlated between the two protocols, due to distortions in the expression ratios (scatter on figure 5c). Amongst the FCE probesets, some still show reasonable nlPv following TwoRA (> 10). Inspection of these genes revealed that they have large expression ratios in the OneRA and moderate ratios in the TwoRA (the median log2 expression ratios was 5.09, 2.52 respectively). By contrast, those FCE probesets with low nlPv (< 10) in the TwoRA have had their log2 expression ratios reduced severely following TwoRA (median log2 ratio in the TwoRA = 0.43).

Interestingly, the latter have, on average, moderate expression ratios in the OneRA (median log2 ratio in the OneRA = 2.8). This is expected since with moderate expression ratios, any reduction would have a greater impact on their significance level. Indeed, looking at the whole population of probesets, out of those with a nlPv between 10 and 20 in the OneRA, only 69% have an nlPv above 10 in the TwoRA, compared to probesets with high nlPv (> 20) in the OneRA where 87% (in agreement with previous results, Table 3) of them have nlPv above 20 in the TwoRA. This suggests that the TwoRA protocol is more suitable with experiments where large differences in gene expression are occurring.

## Discussion

Microarray technology is currently limited by the need for relatively large transcript quantities, which makes it incapable of handling small biological samples. The T7 in-vitro transcription has been widely explored to achieve a linear amplification of targets for microarrays. Much work has considered the effect of linear amplification on the ability to profile gene expression. Although, the reproducibility of such techniques and their fidelity in maintaining absolute levels of expression have been extensively analysed, much less is known about their ability to accurately reproduce differential expression in distinct biological samples. This study gives further insights on the impact of linear amplification using the Affymetrix small sample protocol on expression ratios and differential expression.

Our analysis confirms the high reproducibility of the small sample TwoRA protocol and the occasional failure in its fidelity to maintain the original levels of gene expression. In this study, robust analyses were used to confirm the 3' bias role in signal distortion. Instead of limiting our analysis to control genes, as previously undertaken by other groups [2,4,6,7], evidence was obtained by examination of intensity data from all probesets on the array. In addition to 3'bias, this work has explored the relationship between distortions in signal intensity following TwoRA and the original intensity level prior to TwoRA. The fact that the intensity range is limited by background noise on one end and saturation on the other end implies that intensity may only be shifted by a limited amount. This relationship bears important consequences on the consistency of the TwoRA protocol in amplifying targets with varying intensities across different samples. Thus, the shifts in intensity following amplification will not appear to be equivalent in the two samples if the shift in one of the samples is limited by the range of the scanner. This has the consequence of distorting the expression ratios, as clearly demonstrated by our data.

Unsurprisingly, the statistical significance of expression ratios is only affected when the expression ratio in the TwoRA is reduced to the point where it can no longer be distinguished from noise. Importantly, large ratios are less likely to be critically diminished and more likely to remain significant following TwoRA. This explains why despite the distortions in ratios in our dataset, there was up to 87% agreement in the top significant genes (nlPv > 20) from the TwoRA and OneRA (DRG,SN). On the other hand, less agreement was observed among the less pronounced ratios (69%) since distortions are more critical. This leads us to the important conclusion that TwoRA may affect the statistical significance of genes with moderate expression ratios to a greater extent. Another important finding by this study is that limma performs best at recognising similarity in differential expression between the OneRA and the TwoRA. Thus, the choice of statistics will also affect the quality of information obtained from microarray analysis of amplified samples.

## Conclusion

We conclude that the Affymetix small sample amplification protocol is useful with the following caveats: First, it should be only used when tissue homogeneity is a crucial factor and sufficient amounts of starting material cannot be obtained by any other means. Secondly, target amplification using the small sample protocol appears to be suitable in situations where big differences in gene expression are expected. Fortunately, it is reasonable to expect large differential expressions with experiments characterising different cells within a mixed tissue where amplification of transcript is necessary. Finally, application of such technique in combination with limma statistical analysis is useful in experiments where the ultimate purpose is to experimentally characterise a finite number of targets, for example the top 100 differentially expressed genes.

However, expression data obtained from amplified samples might be less suitable for more comprehensive numerical analysis, for example characterising regulatory networks, due to the problems caused by possible shifts in signal and expression ratios.

## Methods

### - Tissue isolation and RNA extraction

Three pools of tissue were prepared from C57/B6 adult male mice after cervical dislocation or decapitation. Tissue from each animal was dissected within 10 minutes of killing and collected on dry ice. One sample consisted of dorsal root ganglia (DRG) of all spinal levels from 10 naïve mice. Another sample (SA) was the lumbar enlargement of the spinal cord of 5 animals that had undergone a unilateral sciatic nerve transection at mid-thigh level under isoflurane anesthesia 7 days previously. A third sample (SN) was the lumbar enlargement of the spinal cord of 5 normal mice. RNA was prepared using the Trizol method (Invitrogen) followed by a cleanup step using the RNAeasy Mini kit (Qiagen). RNA quality and quantity was measured by microfluidic electrophoresis (Nanochip running on a 2100 Bioanalyzer, Agilent Technologies, Palo Alto, USA). The 28S/18S RNA ranged from 1.3 to 1.4 in all samples. The total amount of RNA was 42.2 μg, 40.1 μg, and 25.3 μg for the DRG, SN, SA samples respectively. The corresponding RNA integrity numbers (RINs) were 8.3, 9.3 and 9.4. RINs larger than 8 are considered to be perfect for downstream applications [37].

### - RNA amplification and chip hybridisation

RNA processing and hybridization followed the Affymetrix methodology. In brief, the Affymetrix standard protocol [8] (in the article referred to as the OneRA protocol) was used to generate three labelled cRNA samples from each tissue pool using 5 μg of total RNA as starting material (Figure 1a). The Affymetrix small sample protocol version 2 [9] (referred to as the TwoRA protocol) that incorporates one extra round of IVT was used to generate 4 labelled samples using 50 ng of starting material from each pool (Figure 1a). The quality of cRNA from all tissue pools and various treatments was assessed using microfluidic electrophoresis. Similar profiles were obtained, although there was a significant shift towards smaller RNA fragments with the TwoRA protocol. Material from the 21 target preparations was then hybridized to MOE430A chips.

### - Data capture and data processing

Scanned images were first inspected for quality control (QC) using a variety of built-in QC tools from the Bioconductor package [10] of R, the open source environment for statistical analysis. QC consisted of visual examination of probe array images, scatter plots from replicates, hierarchical clustering of array hybridizations, RNA degradation plots and MvA plots. Feature intensity values from scanned arrays were normalised and reduced to expression summaries using the GC Robust Multiarray Algorithm (GCRMA) [11,12] implemented as a function in the Bioconductor GCRMA library [13]. Detection calls indicating the presence or absence of signal from each probe set were obtained by processing the raw data with the Microarray Analysis Suite 5.0 (MAS5). To obtain a consensus detection call across replicate hybridizations, a probe set was considered to be present if it received a P (present) detection call from all replicates or n-1 replicates with an M (marginal) call from the remaining replicate. A (absent) detection calls were determined in the same way.

For further analysis, probesets 3' locations were obtained by downloading the MOE430a probe tab files made available by the Affymetrix online support [39]. A probeset location was considered equal to the 3' distance of the probe that is most distal from the 3' end of the corresponding target within the set.

To test for differential expression, two statistical methods were used. The bayesian adjusted t-statistics from the linear models for Micoarray data (limma) package [14,15] and the Z-scores method as described by Quackenbush [16]. With both methods, a multiple testing correction based on the false discovery rate (FDR) was performed.

## Authors' contributions

ID participated in experiment design, performed all numerical analysis and produced the manuscript. LW has had an essential input into the statistical analysis of the data and contributed in writing up the manuscript. CAO contributed advice on the analysis and critically reviewed the manuscript. MK designed and implemented the experimental work, participated in the analysis and the drafting of the manuscript. All authors read and approved the final manuscript.

## Figures and Tables

**Table 1:** Summary of the top 10 discrepant probesets whose (DRG,SN) expression ratios from the TwoRA deviate most from the OneRA.

**ProbeSet ID (Gene Symbol)**	**Log 2 intensity**					
	
	**DRG**		**SN**		**SA**	
	
	**1R**	**2R**	**1R**	**2R**	**1R**	**2R**
1425983_x_at (Hipk2)	0.87	0.89	8.20	0.73	7.79	0.70
1422277_at (Glra1)	2.35	2.40	9.65	2.46	9.87	2.46
1422009_at (Atp1b2)	2.98	2.56	12.73	5.50	12.57	4.49
1450177_at (Ngfr)	8.98	2.63	2.25	2.33	2.23	2.27
1425337_at (Slc12a5)	2.34	2.33	9.89	3.50	9.63	3.54
1425963_at (Cabp7)	1.94	1.98	7.88	2.07	7.80	2.03
1439083_at (Ahi1)	2.14	2.17	7.77	2.05	7.96	2.54
1429566_a_at (Hipk2)	1.62	1.55	11.01	5.60	10.78	5.71
1456637_at (Lrrtm2)	8.57	3.35	3.92	4.00	4.59	4.07
1423669_at (Col1a1)	11.06	5.86	2.76	2.81	2.74	2.74

Listed are gene annotations (probeset ID and gene symbol) and log2 intensities from all biological versus protocol groups.

**Table 2:** Evidence of differential expression for the genes with the 10 most discrepant (DRG,SN) tissue ratios from the OneRA and the TwoRA.

**ProbeSet ID Gene Symbol)**	**Log2 expression ratios**		**Evidence for differential expression**
		
	**OneRA**	**TwoRA**	
1425983_x_at (Hipk2)	-7.33	0.15	Hipk2 encodes Homeodomain-interacting protein kinase 2. On the chip, there are two interrogation sites and in both the results from the OneRA indicates that the gene is more strongly expressed in the spinal cord. In the embryo, Hipk2 is expressed in DRG and spinal cord [24] but it is strongly downregulated prenatally in sensory neurons [25]. Judging from LacZ expression in transgenic mice, Hipk2 is highly expressed in the spinal cord, especially in the motor neurons [26].
1429566_a_at (Hipk2)	-9.40	-4.06	
1422277_at (Glra1)	-7.29	-0.06	Glra1 encodes the alpha1 subunit of the glycine receptor. Although it is found in some neurons of the DRG, it is very strongly expressed in the spinal cord [27].
1422009_at (Atp1b2)	-9.75	2.94	ATP1b2 encodes the beta2 subunit of the Na+/K+ transporting ATPase, which is mainly located in glial cells [28]. It is not found in the DRG using immunohistochemistry [29].
1450177_at (Ngfr)	6.73	0.30	Ngfr encodes the low affinity neurotrophin receptor p75. In the adult it is expressed in many neurons of the DRG [30], but largely absent in the spinal cord [31]. A second probeset (ID: 1421241_at) interrogating the same gene reported similar results i.e. the log2 expression ratio in the TwoRA is reduced (3.62, 0.22 OneRA, TwoRA respectively).
1425337_at (Slc12a5)	-7.54	1.16	Slc12a5 is the Solute carrier family 12 member 5, also known as the Neuronal K-Cl cotransporter 2 (KCC2). It is strongly expressed in neurons of the adult CNS including the spinal cord but it is absent in DRG neurons [32].
1425963_at (Cabp7)	-5.95	-0.09	Cabp7 encodes the calcium binding protein 7, which is strongly expressed in spinal cord [33].
1439083_at (Ahi1)	-5.63	0.12	Ahi1, Abelson helper integration site 1, encodes a cytoplasmic adaptor protein [34]. It is primarily expressed in the CNS including the spinal cord [35]. Mutation of this gene causes Joubert syndrome, a congenital malformation of the cerebellum and brainstem with axonal decussating abnormalities of the corticospinal tract [35].
1456637_at (Lrrtm2)	4.65	-0.65	Lrrtm2 is leucine-reach repeat protein 2, also known as neuronal leucine-rich repeat protein 2. It is found in DRG and spinal cord [36] and on the basis of available in situ hybridisation data appears to have stronger expression in the DRG than in spinal cord [36].
1423669_at (Col1a1)	8.30	3.05	Col1a1, is collagen type 1. It is ubiquitously expressed, but prominent in bone and connective tissue. It is possible that the increase in the DRG sample is due to a contamination during the dissection of the DRG (which were harvested after cutting the vertebral column).

The genes have large expression ratios in the OneRA that are reduced in the TwoRA. The log2 expression ratios are calculated as log2 intensity from the DRG group – log2 intensity from the SN group. Thus, if the ratio is positive, the gene is more expressed in the DRG whilst if negative, the gene is more expressed in the SN.

**Table 3:** Similarity in significance calls from the OneRA and the TwoRA (DRG,SN) comparisons using the Z-scores and limma statistics.

**Number of top significant genes**	**Z-scores**			**Limma**		
	
	**SSS(%)**	**FDR**		**SSS (%)**	**FDR**	
				
		**OneRA**	**TwoRA**		**OneRA**	**TwoRA**
100	59	3.7E-3	1.1E-2	87	5.23E-14	1.72E-14
300	59	7.1E-2	0.11	87	2.08E-12	6.56E-13
500	63	0.201	0.214	87	1.52E-11	5.59E-12

The significance similarity score (SSS) is the percentage of genes common to the OneRA and the TwoRA selected sets of top significant genes.
